# Developing emergency medicine leaders: The AACEM/SAEM chair development program at 10 years

**DOI:** 10.1111/acem.70034

**Published:** 2025-03-27

**Authors:** Brian J. Zink, Susan A. Stern, Prashant Mahajan, Kayla Roseen, Apoorva Belle, James Cranford

**Affiliations:** ^1^ Department of Emergency Medicine University of Michigan Medical School Ann Arbor Michigan USA; ^2^ University of Washington School of Medicine Seattle Washington USA; ^3^ Society for Academic Emergency Medicine Des Plaines Illinois USA

## Abstract

The AACEM Chair Development Program (CDP) provides emergency medicine (EM)‐focused leadership training for academic chairs and those interested in becoming EM chairs. The CDP began in 2014.This report describes the CDP second 5‐year cohort from 2018 to 2023. A total of 102 participants completed the program during this time period with increased enrollment of women leaders. Seventeen participants who were not chairs at entry have become EM chairs. Quantitative and qualitative data based on a survey of participants demonstrate continued highly favorable assessment of the CDP and likelihood to recommend it to others. The CDP remains a popular and successful training experience to develop leadership skills, foster a leadership network, and prepare EM leaders for academic chair positions.

## BACKGROUND

The Association of Academic Chairs of Emergency Medicine (AACEM) and the Society for Academic Emergency Medicine (SAEM) sponsor a chair development program (CDP) that was originated by the AACEM Executive Committee in 2013. The CDP is an emergency medicine (EM)‐focused leadership training program that addresses fundamentals of leadership for aspiring and early chairs of academic departments. This program is unique in that while it tackles a broad range of common leadership topics, it also addresses many that are specific to EM. An AACEM past president developed the program that launched in 2014, and he served as its founding director. A codirector was added in 2018. The first 5 years of the CDP were reviewed and described in a previous publication.^1^ The year‐long training format was developed based on the original survey of EM chairs in 2013 that indicated that they would have benefited from this type of program more than a single workshop. Chairs felt that the chief benefit of this approach was development of a network of peer mentors for emerging leaders.

This report describes the characteristics, perceived effectiveness, and impact of the CDP for EM leaders who completed the program between 2019 and 2023. We review the curriculum, report the demographics and characteristics of participants, and discuss the results of a survey of participants who completed the program. Additionally, we compare and contrast the characteristics of 2019–2023 cohort of participants with those from the first 5 years of the CDP.

## CDP DESCRIPTION

The CDP is advertised each year on the AACEM and SAEM websites and in virtual newsletters that invite developing leaders who are early chairs or interim or acting chairs or who may pursue chair positions in the coming years to apply for the CDP candidates who are chairs or interim chairs complete a simple application document and are accepted into the program. Those who are in nonchair leadership roles complete the application form and submit their curriculum vitae and a support letter from their chair. Applications are reviewed by codirectors who decide on acceptance to the program. While there are no specific criteria for acceptance into the CDP in terms of academic rank or previous leadership experience, those who are not accepted are generally viewed to be too early in their careers to receive maximum benefit from the CDP at that stage. Tuition for the CDP has remained stable compared to inflation with the goal to keep it affordable for participants and their departments and to cover the costs of the program. All the EM leaders who serve as presenters, invited speakers, or panelists in CDP sessions volunteer their time to teach in the program. One non‐EM instructor receives a modest honorarium for their session.

The gender and ethnic diversity of the initial CDP classes were less than what the founders had hoped for. After discussions with AACEM and SAEM leadership the Academy for Women in Academic Emergency Medicine (AWAEM; 2017) and the Academy for Diversity and Inclusion in Emergency Medicine (ADIEM; 2018) each established a scholarship to support one position per year. AACEM established the memorial Chris King Scholarship in 2018 to support one position as well. Class size was intentionally increased from an average of 15 per year to 18 to 20 positions per year starting in 2018 to meet increasing demand for the program.

The basic curriculum for the year‐long CDP has remained largely consistent since its inception. Minor modifications have been made based on formal and informal feedback from the participants and the evolving issues and challenges within EM. CDP participants provide feedback through an evaluation form that includes narrative comments for each session and a year‐end evaluation. Based on this CDP sessions are revised, added, or sometimes removed. An example of the CDP curriculum for 2023 is provided in Table 1.

**TABLE 1:** CDP curriculum for the 2023 class.

First session—January—in‐person. Preceded by a mixer with the outgoing CDP class:
Introduction to CDP (1 h)—group introductions, review of program.
Mission, Vision, Values (1.25 h)—how to collaboratively create the MVV for your department.
Innovation Genome Exercise (1 h)—Prework and exercise, competing values framework to assess individual and departmental values and culture.
The New Chair in Town (1.25 h)—Approach and strategies for the first 100 days of a chair term.
The A Team (1 h)—Assessing, recruiting, and building an academic EM faculty group; defining roles, assignments, and programs.
Second session—March, in conjunction with the AACEM annual retreat
EM Finances 101 (1.5 h)—Basics of accounting, budgeting, and funds flow in an academic department of EM, health system, and medical school.
Philanthropy in EM (1 h)—Understanding and building development funding and philanthropy in academic EM
Building the EM Departmental Team (1 h)—How to work effectively with a lead departmental administrator; how to construct the overall department leadership team.
Return on Investment (2 h)—How to make the case for funding EM clinical, educational, and research priorities to the medical school and health system.
Research; Yes? No? How much? (1.5 h)—How to build a successful, right‐sized research program for an academic EM department.
“Hot Topics” (1 h)—Open forum question/answer and discussion for CDP participants and faculty.
Third session—May—CDP participants are automatically enrolled in the SAEM Leadership Forum
Finding Meaning (1 h)—Identifying strategies to create an environment of personal fulfillment during an academic career in EM.
Physician Wellness: A Holistic Approach to Burnout Prevention at the Departmental Level (1.5 h)—How to prioritize well‐being using a multifaceted strategy that will enable physicians to thrive both personally and professionally.
Creating Space So Everyone Values Diversity, Inclusion, and Equity (45 min)—Building an inclusive culture and improving team engagement, by creating diverse and inclusive teams.
Managing Conflict with Win–Win Strategies (1 h)—Methods for successfully addressing conflict with cases from academic EM.
Supporting and Reigniting the Academic Mission (1.5 h)—Successful leaders offer pearls and personal anecdotes with important take‐home messages for aspiring leaders.
Professionalism Issues (1.5 h)—Case‐based and small group discussion to discuss and highlight best practices to address lapses in professional conduct.
Coaching—the leadership tool of the 21st Century (1 h)—Defining the essential skills of coach‐like leadership, illustrate these skills through a live demonstration, and allow participants to practice coach‐like skills with a peer.
Monthly virtual sessions (June–December). Attendance at 80% of these sessions is required, and sessions are recorded for those who miss sessions
Effective Feedback (1 h)—How to provide timely, meaningful feedback as a chair to department leaders, faculty, and institutional colleagues.
Change Management (2 h)—Understanding how to manage and communicate change efforts in academic EM.
EM Regional Networks (1 h)—Review of an effective regional EM network and tips on how to build an effective multi‐ED department as a chair.
Chair Role in EM Operations (1.5 h)—Understanding how a chair can work with the ED team to assess and improve ED clinical operations and metrics.
Coding and Billing (2 h)—Primer on the basics EM chairs in ED clinical coding, billing, and funder threats and opportunities.
Chair Challenges (1.25 h)—Case‐based review and discussion of common professional and personal issues in the chair role.
Chair Leadership for Financial Success (1.5 h)—Second session provided by an experienced EM administrator on academic EM revenue and finances with examples.
Professionalism and Faculty Conflict (1.5 h)—How to effectively address faculty professional misconduct and difficult personnel issues as a chair. Case‐based.
Fifth session—January
Chair Time Management (2 h)—Review of best practices for managing schedule and time and preventing overload for an EM chair.
Media Communications (2 h)—Participatory workshop on media strategy and tactics for an academic EM chair. Taught by an experienced media consultant and former reporter.
Leadership Resilience (1.5 h)—Discussion‐based session with examples and case studies on how to deal with the stressors of being an EM chair.
The Chair Hunt (1.5 h)—How to identify chair opportunities (internal and external) evaluate the position and interview for an EM chair job.
Recap of CDP—Lessons (1 h)—Review of all sessions with key take‐home points.
Dinner with Incoming CDP Class—Networking and discussion.

*Note*: The general approach for sessions is a limited didactic portion followed by a question/answer period and facilitated discussion with CDP participants. PowerPoint slides and any reading materials are distributed to the class. Panels of current chairs are utilized in several sessions. Total number of hours for CDP formal sessions is 41.

Abbreviation: CDP, chair development program.

Participants are required to attend all in‐person sessions and 80% of virtual sessions. Virtual sessions are recorded and must be viewed later if participants cannot attend those sessions. Participants receive a certificate of completion at the end of the CDP.

Prior to 2020, all sessions were conducted in person at specific CDP meetings or in conjunction with EM national meetings. The COVID pandemic necessitated moving the 2020 class to all virtual (Zoom platform) meetings after their initial in‐person all‐day session in January. In 2021, because of the convenience and lower cost of virtual sessions the CDP structure was changed to include a combination of in‐person and virtual sessions. The program supports 4 day‐long in‐person sessions as follows: the initial January session, a session in conjunction with the AACEM retreat, the SAEM leadership forum, and a final session in January of the following year. The remainder of the sessions are conducted virtually via Zoom (approximately 30% of the content). This balance of in‐person and virtual sessions allows for maximum participation but continues to promote the goal of camaraderie and relationship development among each cohort.

## METHODS FOR SURVEY OF PARTICIPANTS

A Qualtrics, web‐based survey was used to generate both qualitative and quantitative data from the 2019–2023 5‐year cohort participants to evaluate the program. The survey was adapted from a previous version used for the first 5‐year cohort (2014–2018) to better reflect the experiences and challenges of the new cohort (2019–2023). The revised questionnaire included both closed‐ended (Likert scale) and open‐ended questions aimed at capturing detailed feedback on the leadership training, engagement across different learning formats (virtual, hybrid, and in person), and the overall impact of the program on participants’ leadership roles.

The final survey consisted of 20 items, including demographic questions, program evaluation components, and items related to participants’ career progression post‐CDP. The survey for this cohort added three questions on whether participants had pursued chair positions where they were not selected for the position, whether they had participated in other leadership development programs, and if so, how the CDP may have enhanced that training. The Likert‐scale responses ranged from 1 (strongly disagree) to 5 (strongly agree), and open‐ended questions allowed for additional qualitative insights. The full survey is provided in Appendix S1.

The study was determined to be not regulated as human subjects research by the University of Michigan Institutional Review Board (HUM00254533) as it involved minimal risk and focused on program evaluation.

### Participants and survey distribution (data collection)

The project manager (AB) was responsible for programming the survey into Qualtrics and creating unique survey links for each participant. Survey distribution occurred over a 3‐month period, with reminders sent every 10 days to those who had not yet completed the survey. Of the 102 targeted participants, valid email addresses were available for 99 individuals; three email addresses were either inactive or unreachable.

### Data management and statistical analysis

All survey data were stored securely in the Qualtrics platform, and data were deidentified prior to analysis. The project manager was responsible for data management, including monitoring response rates and ensuring data security. Data were exported from Qualtrics in CSV format for further analysis. Access to the raw data was restricted to the project manager and CDP codirectors.

Quantitative analysis of the data was conducted by a statistician (JC) to summarize demographic characteristics and response patterns across the survey items. Descriptive statistics, including frequencies, means, and standard deviations (SDs), were calculated for the Likert‐scale questions. All statistical analyses were conducted using the SPSS statistical software package (Version 29.0XX).

Qualitative analysis of open‐ended responses was conducted with assistance from the University of Michigan's GPT (UM‐GPT) tool, a natural language processing model that is meant for text analysis, to qualitatively facilitate the identification of recurring themes and key insights. The authors provided UM‐GPT with a structured prompt that included the original survey question, so that the AI‐identified themes were aligned with the intended scope of the responses. The model processed the text, identified the recurrent ideas or phrases, and grouped them into preliminary themes. These preliminary themes created by the AI were reviewed separately by the authors (BZ and AB) who systematically audited the relevance, coherence, and contextual accuracy of the themes. Responses were thematically analyzed to extract information on the strengths, areas for improvement, and perceived impact of the program.

General descriptive data for all CDP 2019–2023 participants, including gender, academic position at entry to the CDP, and work institution, were available through AACEM/SAEM records. Web‐based searches were conducted to determine whether CDP participants who did not complete the survey had become chairs after enrollment in the CDP.

## RESULTS

The data reported in this section are derived both from application and from enrollment data for all 102 CDP 2019–2023 participants and from the postparticipation survey. The percentage of CDP 2019–2023 participants who responded to the postparticipation survey was 70%, down from the 94% response rate for the CDP 2014–2018 classes despite the email reminders and targeted emails to nonrespondents.

Over the first 10 years of its existence, 182 physician leaders completed the CDP, with 80 in the first 5‐year cohort and 102 in the 2019–2023 cohort. Eighty‐five to 90% of applicants were accepted each year into the CDP in this 2019–2023 cohort. There was an increase in more junior applicants in the 2019–2023 cohort. The small number of nonacceptances was based on applicants judged as being too early in their careers or because they did not fully complete the application packet. The CDP 2019–2023 participants and their gender identities are summarized in Table 2. The percentage of participants who identified as women increased from 16% in the 2014–2018 cohort to 43% in the 2019 to 2023 cohort. The percentage of women in the 2019–2023 cohort increased progressively over the 5 years—from 39% in the CDP 2019 class to 59% in the CDP 2022 and 2023 classes. Overall, there were 13 women in the 2014–2018 cohort and 45 in the 2019–2023 cohort. The number of participants who identified as underrepresented in medicine (URiM) was not reliably tracked at entry to the program. However, of the 70 respondents who completed the postparticipation survey, 8.6% indicated that they were Black or African American, and 11.4% indicated that they were Hispanic. Academic medical institutions from across the United States were widely represented in the CDP 2019–2023 cohort with some institutions having more than one participant over the 5 years, but none had more than five participants. Fifty‐eight different medical schools were represented in this 5‐year cohort. Fifteen of 102 (15%) participants came from nonuniversity academic medical centers that had medical school affiliations, while the rest were faculty at university‐based medical school departments of EM.

**TABLE 2:** CDP class sizes and male/female composition, 2019–2023.

	CDP '19	CDP '20	CDP '21	CDP '22	CDP '23
Men	13	12	14	11	9
Women	7	8	12	9	9
Total	20	20	26	20	18

Abbreviation: CDP, chair development program.

Overall, participants’ academic positions at the time of CDP enrollment were similar in the second cohort to the first cohort, with 50%–60% each year being vice chairs or associate chairs in academic departments of EM. The percentage of enrollees who were chairs or interim chairs at entry to the program was 20% in the 2019–2023 cohort compared with 19% in the 2014–2018 cohort. There were more division directors, residency program directors, and clinical emergency department directors in the 2019–2023 cohort than the 2014–2018 cohort. At entry to the program 50% of CDP 2019–2023 survey respondents were associate professors and 43% were professors. A higher percentage of men (47.1%) than women (30.6%) indicated that their academic rank was professor (*χ* ^2^(1) = 2.0, *p* = 0.16.) By the time of completion of the survey in 2024, the overall percentage of professors in this group of survey respondents had increased to 66% but was lower in women (61%) than in men (70%). The percentage of survey respondents who were chairs at the time of the post‐CDP survey was lower among women (22%) than men (38%).

The percentage of participants in this cohort who have become chairs during or after completing the CDP was 17%. In the previous 5‐year cohort it was 29% at a similar time point. Some survey respondents assumed non‐EM chair leadership positions with five reporting that they were associate deans for graduate medical education (GME) at their institutions and/or designated institutional officers (DIO) for GME. One reported being an associate DIO and another reported being an assistant dean for clinical applications.

For those survey respondents who were not chairs at the time of the survey, 25 of 70 (36%) responded “yes” to the question: “Have you pursued a chair position or positions where the result was that you were not selected for the position you had applied for?” A higher percentage of men (41.2%) than women (30.6%) indicated that they had an unsuccessful chair application, but this difference was not statistically significant (*χ* ^2^(1) = 3.4, *p* = 0.18).

The CDP was highly rated by participants. Almost all (96%) respondents agreed or highly agreed with the statement “I would recommend the leadership training of the CDP to others” (Likert‐scale mean ± SD 4.7 ± 0.6). For survey respondents who were currently chairs, 100% responded that they agreed or highly agreed with the following statement: “The CDP was effective in preparing me for the role of a chair/improving my performance as a chair” (Likert‐scale mean ± SD 4.5 ± 0.5).

The qualitative analysis provided additional information and perspectives on the program. Table 3 summarizes the questions, themes from responses, and representative responses. Many respondents valued the in‐person networking, education on the specifics of the EM chair role and duties, leadership learning, and financial management sessions. As of January 1, 2025, 38% (52/134) of active chairs in the AACEM community had completed the CDP.

**TABLE 3:** Qualitative survey responses summary.

Survey question	Response themes	Representative responses
Please describe the role, if any, the CDP made in choosing your career path and decision making, specifically regarding a chair position	Positive influence on interest/desire to become a chair Role in better understanding and preparation Impact on confidence and skills development	“CDP fortified my desire to pursue a chair position.” “CDP explained the wide‐ranging aspects of a chair position.” “Helped me build confidence in my ability to successfully lead an academic department.”
Please list up to three aspects of the CDP that you found most valuable	Networking and relationships Financial and budget management Leadership and management skills	“Networking and interaction with leaders in different health systems.” “Financial discussion; how to get people on ‘the bus’.” “Creative problem solving.”
Are there topics that were not taught in the CDP that would you like to see included in the program?	CV and application support Diversity, equity, and inclusion (DEI) HR and personnel management	“Maybe would have helped to get some CV feedback.” “More on managing diversity and inclusion policies, practice, and realities.” “Maybe more on HR/employment law.”
Please list up to three ways the CDP could be improved	In‐person engagement Structured networking and mentorship Access to content and curriculum	“The in‐person sessions were the highest yield. Maximizing those as much as possible would be desirable.” “Some continued mentorship with faculty of CDP or graduates.” “Consider a CDP alumni list with access to current CDP lectures in an on‐demand format.”

Abbreviation: CDP, chair development program.

## DISCUSSION

In more than a decade of its existence, the CDP has been highly effective in achieving its goal of enhancing the leadership skills of new and aspiring academic EM chairs as evidenced by both quantitative and qualitative data. It has served as a key pipeline for leadership development in EM and non‐EM leadership positions in academic medicine.

The CDP is the only leadership development program in EM that is focused on department chairs and those who intend to become chairs. Some other medical specialty organizations, including internal medicine, urology, neurosurgery, neurology, radiology, and ophthalmology, have longitudinal (1–2 years) development programs for leaders who are not early career, but these are not specifically oriented toward chairs.^2^, ^3^, ^4^, ^5^, ^6^, ^7^ The association of departments of family medicine has an annual workshop that is aimed at a small group of new chairs who are provided consultation and case‐based mentoring.^8^ A pediatrics chair organization provides two longitudinal leadership development programs that are focused on mentoring relationships for new and prospective chairs.^9^

In its second 5 years (2019–2023) the CDP increased its annual enrollment, is training more women, and recently has enrolled more leaders who identify as URiM. Since the inception of the program, approximately one in five CDP participants have been chairs or interim chairs at entry to the program. Vice chairs and associate chairs continue to make up the majority of participants but faculty in other leadership positions such as division directors and residency program directors are increasing. The variety of leadership roles and perspectives of this diverse group of CDP participants contributes to the value of the training experience in the group discussions during CDP sessions and in the peer‐to‐peer mentoring that arises from the cohort. The codirectors are aware of numerous instances where CDP graduates have consulted each other when applying for chair positions or when dealing with issues as chairs.

The percentage of women participants increased substantially in the 2019–2023 classes. This is felt to be due to the CDP codirectors reaching out to AWAEM, the formation of the AWAEM CDP scholarship, and discussions initiated by the codirectors with women EM chairs about ensuring that women leaders were aware of and applying for the CDP. Women were less likely to be in chair positions at entry into the CDP. However, fewer women reported that they had been unsuccessful when seeking chair positions post‐CDP when compared to men. The reasons for this will require further assessment and analysis.

The curriculum of the CDP has changed slightly over the past 5 years, but as the great majority of sessions have been favorably evaluated by participants, most have continued with some revisions. The primary change has been the transition to having more sessions conducted virtually rather than in person. While participants noted the convenience of virtual sessions, they also described the importance of networking for their leadership development and that this occurred more readily in the in‐person sessions.

The effects of the COVID pandemic were seen in the 2020 CDP class, where participants noted the drawbacks of having entirely virtual sessions after their first in‐person session. The most noted issue was the difficulty with networking with classmates and forming relationships that would extend beyond the CDP year. The value of in‐person networking is weighed against the convenience and lower cost of virtual sessions. Given the concerns raised in the qualitative feedback from the 2020 experience with an all‐virtual curriculum, the in‐person components of the CDP were reinstated in 2021 and continue with the current curriculum. To facilitate networking and promote ongoing relationships among each cohort, a listserv is created for each class. Informal feedback has identified this as a significant benefit.

The CDP continued to be rated very favorably by participants, with almost identical Likert‐scale scores for the “recommend to others” question (4.75 for the 2014–2018 cohort and 4.7 for the 2019–2023 cohort) and “effective in improving my performance as a chair” (4.65 for the 2014–2018 cohort and 4.5 for the 2019–2023 cohort). The addition of a more in‐depth qualitative analysis of participants comments shed additional light on the best aspects of the CDP training as well as areas for improvement. This revealed that participants most highly valued CDP networking and relationships, financial and budget management, and leadership and management skills. Many reported that the CDP helped them better understand the responsibilities and processes involved in a chair position, thereby preparing them for such roles. Others noted that they had increased confidence in their ability to be a chair and/or pursue chair positions in the future.

While many participants in this cohort noted that the CDP reinforced their decision to pursue a chair position, a small number noted that the additional knowledge of what a chair job entails prompted them to reconsider their career goals and realized a chair position was not consistent with their career goals. Program leaders view this reflection by some CDP participants as positive, facilitating meaningful self‐reflection and more informed career decisions.

The 2019–2023 CDP cohort identified some areas for improvement or expanded coverage in the CDP including more attention to financial management, human resources issues, additional leadership skills, and more practical and personalized support like curriculum vitae preparation and effective interviewing for a chair position. The program will continue to be modified by the codirectors to address these opportunities.

With almost four out of 10 active chairs in academic EM having completed the CDP, the reach of the program is substantial. However, some leaders who become EM chairs have not completed the CDP or choose not to enroll early in their chair term. The reasons for this deserve further inquiry and study. A goal of the program directors is for all new chairs to have the benefit of CDP training as they embark on their new leadership journey.

## CONCLUSIONS

Over the past 10 years, the AACEM/SAEM Chair Development Program has been highly effective in achieving its goal of enhancing the leadership skills of new and aspiring academic emergency medicine chairs. This program addresses a broad range of general leadership topics from the unique lens of emergency medicine. Based on participant feedback, the mixture of case‐based learning, panel discussions, and exposure to and interaction with a range of leaders within emergency medicine as well as networking opportunities have been important factors in the success of this program. The 2019–2023 chair development program cohort was larger, trained more women leaders, and was again rated very highly by participants.

## AUTHOR CONTRIBUTIONS

B.J.Z conceived of the manuscript; collected the demographic data for the 5 CDP classes; obtained IRB exempt approval; helped to design the survey; sent the survey from his email; reviewed the data and survey results; wrote the manuscript with significant help from the co‐authors. S.A.S. helped to plan the manuscript; helped in designing the survey; reviewed data and survey results; had a significant role in writing and editing the manuscript. P.M. helped to plan the manuscript; reviewed the data and survey results; had a significant role in writing and editing the manuscript. A.B. helped to plan and conduct the survey, organized and analyzed the qualitative data, had a significant role in writing the methods section of the manuscript. K.R. helped to identify survey participants, provided data from SAEM and AACEM on CDP participants; reviewed the manuscript and tables and provided feedback on the manuscript. J.C. managed survey data, conducted the quantitative statistical analysis of survey results; assisted in writing the methods and results section of the manuscript.

## FUNDING INFORMATION

SAS reports grant money to University of Washington to conduct research conceived and written by SAS from University of Washington. None of this research relates to the submitted manuscript. PM reports grant money to University of Michigan to conduct research conceived and written by PM from University of Michigan. None of this research relates to the submitted manuscript. BJZ, AB, and JC report no grant funding.

## CONFLICT OF INTEREST STATEMENT

The authors declare no conflicts of interest.

## Supporting information




Data S1.

## Data Availability

The data that support the findings of this study are available from the corresponding author upon reasonable request.
