# Supplementary material for: Developing emergency medicine leaders: The AACEM/SAEM chair development program at 10 years
Source: Acad Emerg Med. 2025 Mar 27;32(6):681–7. doi: 10.1111/acem.70034 (PMC12171658; doi:10.1111/acem.70034)
Supplement: Supplementary file 1 — Data S1. [file ACEM-32-681-s001.pdf]

Default Question Block

This year marks the 10-year anniversary of the AACEM Chair Development Program (CDP). As we did after the first 5 years of the program, we are surveying the graduates from years 6 through 10. We seek qualitative and quantitative data to characterize the impact of the program, its strengths and ways to improve it. It is essential to get a robust response from you, who engaged in the program. As before we intend to submit a manuscript describing the evolution of the CDP. All data in that manuscript will be de-identified. Please take a few minutes to fill out this survey.

This survey is deemed to be not regulated by the University of Michigan's Institutional Review Board (HUM00254533).
If you have any questions or concerns, please contact the project manager, Apoorva Belle, at Belleap@med.umich.edu.

Many thanks,
Brian Zink and Susan Stern
CDP Co-Directors

Please fill out the form below

Email Address	
Last Name	
First Name	
Gender	
Race	

Your CDP class year

- ◯ 2019-2020
- ◯ 2020-2021

- ○ 2021-2022
- ○ 2022-2023
- ○ 2023-2024
- ○ [ ] Other

Institution at time of CDP enrollment

[ ]

Your current role

[ ]

Your current academic rank

- ○ Assistant
- ○ Associate
- ○ Professor
- ○ Endowed Professor
- ○ [ ] Other

Are you currently a chairperson?

- ○ Yes
- ○ No
- ○ [ ] Other

Have you pursued a chair position or positions where the result was that you were not selected for the position?

- ○ Yes
- ○ No
- ○ [ ] Other

If you are not a chairperson, will you pursue a chair position?

- ○ Yes
- ○ No
- ○ [ ] Undecided

If you are currently a chairperson, respond to the following statement: The CDP was effective in preparing me for the role of a chair / improving my performance as a chair.

1 Highly Disagree	2 Disagree	3 Equivocal	4 Agree	5 Highly Agree
○	○	○	○	○

If you are not currently a chairperson, respond to the following statement: The CDP was effective in improving my performance as a leader in my current role.

1 Highly Disagree	2 Disagree	3 Equivocal	4 Agree	5 Highly Agree
○	○	○	○	○

I would recommend the leadership training of the CDP to others.

1 Highly Disagree	2 Disagree	3 Equivocal	4 Agree	5 Highly Agree
○	○	○	○	○

Please describe the role, if any, the CDP made in choosing your career path and decision- making, specifically regarding a chair position.

Please list up to three aspects of the CDP that you found most valuable.

Are there topics that were not taught in the CDP that would you like to see included in the program?

Please list up to three ways the CDP could be improved.

Have you participated in other leadership development programs?

- Yes
- No
- Other

If yes, please list:

If yes, please provide any unique features of the CDP and how it further enhanced the training you may have already received.

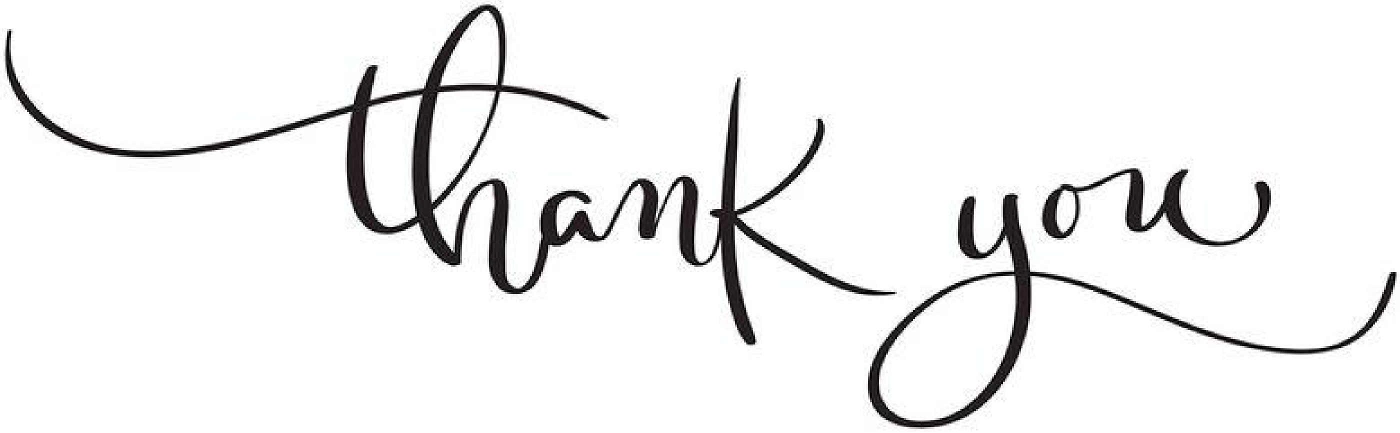
thank you
